# Supplementary material for: Are carbon emissions trading and green financial instruments synergistic? -Comprehensive quantitative research based on content analysis
Source: PLoS One. 2024 Mar 7;19(3):e0298601. doi: 10.1371/journal.pone.0298601 (PMC10919601; doi:10.1371/journal.pone.0298601)
Supplement: S1 Data — (ZIP) [file pone.0298601.s001.zip › Data/地级市Z_F工作报告.zip!/║╙─╧/┐¬╖Γ╩╨/└┤╫╘╣1⁄2╓┌║┼í╛┬φ┐╦╩2╛▌═°í┐_2016.docx]

各位代表：

　　现在，我代表市人民政府，向大会作政府工作报告，请予审议，并请各位政协委员和列席人士提出意见。

　　一、2015年及“十二五”工作回顾

　　2015年是“十二五”规划的收官之年，也是我市经济社会发展极不平凡的一年。面对严峻复杂的经济形势，市政府在省委、省政府和市委的坚强领导下，在市人大、市政协的监督支持下，团结带领全市人民，坚持“1234”总体思路，坚持“好中求快”总基调，抓培育、扩增量、上项目、建平台、优环境、惠民生，圆满完成了市十四届人大二次会议确定的目标任务。

　　全市生产总值完成1604.8亿元，增长9.4%，居全省第3位，创历史最好位次；规模以上工业增加值578.2亿元，增长10.3%，居全省第2位；一般公共预算收入108.3亿元，增长12.6%，居全省第3位；社会消费品零售总额747.1亿元，增长12.9%，居全省第7位；固定资产投资1324.5亿元，增长16.6%，居全省第11位；预计城镇居民和农村居民人均可支配收入分别达到23400元和10500元，增长9%和10.5%；居民消费价格涨幅2.1%。一批事关发展全局和人民群众切身利益的大事要事实事取得重要进展，发展基础更加坚实，发展支撑更加有力，发展前景更加广阔。

　　——新兴副中心城市定位更加清晰。经过多年来坚持不懈努力，开封在全省发展格局中的地位更加重要，省委《关于河南省国民经济和社会发展第十三个五年规划建议》提出，“推动开封建设新兴副中心城市，建设开港经济带、郑汴产学研结合示范带、沿黄生态带，打造郑汴一体化升级版”，进一步明确了开封发展的战略定位和目标方向。

　　——“双+”驱动厚植优势日益凸显。积极推进“互联网+”行动，在全国首先提出“文化+”理念，出台《“互联网+”三年行动计划》和《“文化+”行动实施意见》，分别推出十个工作专项，“互联网+”举措精准有力，“文化+”内涵不断丰富，“双引擎”“双动力”引领城市转型发展步伐加快。

　　——融入“一带一路”战略抢占先机。在全国率先发起成立“一带一路”城市旅游联盟，吸引国内外33个城市踊跃加入，搭建了开放发展新平台，开封旅游和宋文化品牌更加响亮，城市知名度和影响力进一步提升。

　　——创新驱动形成转型升级新动能。出台加快发展众创空间、推进创业创新系列政策措施，加强科研机构、创新基地建设，新建新型研发机构6家，建成众创空间10家，吸引众多高层次人才在开封创业发展，大众创业、万众创新氛围日益浓厚。

　　——城市品位和美誉度持续提升。成功创建国家卫生城市、国家园林城市、省级旅游标准化示范城市，获得全国文明城市提名。顺利通过第七次全国双拥模范城验收。2015中国（开封）清明文化节、中国开封第33届菊花文化节、豫台经贸洽谈会、“文化+”高端对话等活动精彩纷呈。

　　一年来，我们主要做了以下工作：

　　（一）稳增长、保态势，经济保持健康较快发展。打出政策组合拳。先后出台《促进经济平稳健康较快发展若干意见》《财政支持稳增长若干政策措施》《促进房地产市场持续健康发展意见》等16项政策措施，实施稳增长保态势二十个重大专项，加强和完善企业服务，针对规模以上企业、“双50”企业，“一企一策”进行帮扶，确保精准发力，稳住基本面。实施金融服务“双计划”。加强银企对接，达成资金支持协议230.4亿元；设立1.7亿元企业还贷周转金，使用规模达4.5亿元；积极帮助企业上市融资，牧宝车居等5家企业在新三板挂牌上市。人民币存贷款余额分别达到1452亿元和1018亿元，较年初增加179亿元和153亿元，增幅均居全省第3位。促进房地产业健康发展。加快房地产去库存化，市区商品房销售面积、销售金额分别增长43%和40.3%；房地产开发投资168.2亿元，增长23.7%，增幅居全省第4位，房地产销售、投资逐步回暖。

　　（二）抓培育、调结构，产业结构加快转型升级。预计，一二三产业增加值分别增长4.2%、9.3%和11.5%，三次产业结构为17.8∶41∶41.2，第三产业比重首次超过第二产业比重，率先在全省形成“三二一”产业发展格局。大力发展现代农业。建成高标准粮田40万亩，新增改善有效灌溉面积35万亩，农业机械化率达到81%，粮食总产291.7万吨，增长4.7%，实现“十二连增”。培育现代农业产业化集群16个，农业产业化龙头企业达到163家。新增标准化规模养殖场60个，肉蛋奶产量稳定增长。促进工业提质增效。全市规模以上工业企业实现主营业务收入2333亿元，增长8.2%，其中主营业务收入超10亿元企业达到29家。工业投资完成707.6亿元，同比增长19.0%，增幅居全省第3位；集聚区内494家规模以上工业企业完成增加值350亿元，增长12.9%，拉动规模以上工业增加值增长8.9个百分点。产业集聚区基础设施进一步完善，带动能力进一步增强，基本完成与所在乡镇套合。汴西产业集聚区获省十先产业集聚区。着力发展服务业。旅游业持续升温，全市旅游接待量4492万人次，实现旅游综合收入245亿元，分别增长13.2%和27.3%。一批“文商旅”融合项目相继完工投入使用，新业态、新模式发展迅速，电商企业达到1000多家，电商商户3000多家。服务业“两区”建设步伐加快，鼓楼、杞县特色商业区分别获省十强、十快特色商业区。商贸、邮政、电信、交通运输业健康发展，服务业增加值完成661.8亿元，增长12.3%，增幅居全省第3位。加快创新驱动步伐。鼓励支持企业技术创新和技术引进，加强与河南大学等本地高校合作，推进与清华大学、中科院上海高等研究所等6家高校及科研机构战略合作。引进院士、国家“千人计划”专家等高层次人才8人，建成院士工作站5个，新培育高新技术企业10家，中小微科技型企业达到302家，科技支撑和引领能力进一步增强。

　　（三）上项目、强支撑，发展后劲持续增强。盯紧抓牢重点项目。“329”工程累计完成投资907亿元；24个省级A类重点项目完成投资175.6亿元；57个市级领导分包项目完成投资233.5亿元。积极主动谋建项目。在华夏大道、开封西湖、建业铂尔曼酒店等项目顺利建成，商登高速、机西高速（开封段）建成通车，郑徐高铁开封北站、德豪光电科技产业园等项目扎实推进的同时，新谋划启动开港大道、郑开城际铁路延长线、涧水河、绿地中部创客天地、智慧城市三中心等一批重大项目。面向全国发布首批30个PPP项目，5个进入执行阶段。完善项目推进服务机制。实施重大项目领导分包、县区会商、联席会议等“六项机制”，健全“招商链、审批链、推进链、保障链”全链条服务，成立市委市政府督查局和市企业投诉中心，加大项目督导力度，狠抓项目跟踪推进。绘制“两图”，编织“两网”，基本实现市、县、乡（镇）、村（社区）四级审批联网，推行“一站式”政务服务，企业和群众办事更加便捷。

　　（四）促改革、扩开放，发展动力活力进一步释放。全力推进改革。深化行政体制改革，新一轮政府机构改革全面完成，市政府工作部门全部建立权力清单和责任清单；推行“三证合一”商事登记制度；全面取消非行政许可审批，行政审批事项由182项精简到129项。深化农村改革，334.7万亩农村土地承包经营权确权登记基本完成，宅基地和集体建设用地使用权确权登记发证有序推进。深化医疗卫生体制改革，实施基本药物零差率销售，县级公立医院综合改革全面启动。机关事业单位工资和养老保险制度、事业单位分类改革扎实推进，交通运输综合执法体制、党政机关公务用车制度改革顺利完成，国有企业、投融资、价格、食品药品监管、司法体制等改革取得新进展。持续扩大开放。建立四个招商基地，完善招商引资“六项机制”，成功举办上海产业合作推介会、京津区域经济技术合作洽谈会等重大招商活动，全市签约项目168个，签约资金1183亿元；实际到位省外资金450亿元，增长10.7%；实际利用外资5.6亿美元，增长20.6%；进出口总值增长3.1%。成立市口岸办，有序推进河南自贸区（开封片区）申报工作。全面启动城市旅游联盟合作，加强与友好城市交流，对外开放迈出坚实步伐。

　　（五）优环境、重协调，城市品位显著提升。加强生态环境保护。扎实推进节能减排，化学需氧量、氨氮、二氧化硫排放量完成省下目标。严守生态红线，实施蓝天、碧水等工程，全市空气质量优良天数222天，居全省第2位；地表水水质平均达标率93.8%，继续位居全省前列。加强林业生态市建设，完成造林9.4万亩。改善新老城区面貌。投资30亿元，加快示范区基础设施建设，中意湖、“三站三馆两中心”等CBD周边项目加快推进，开封西湖形成靓丽景观，示范区形象显著提升。加快老城区改造步伐，完成棚户区、城中村改造164.8万平方米，拓宽改造内环路、内顺城路、魏都路等一批城市道路，环城墙公园、御河水系等城市景观带建设扎实推进，停车场、污水及垃圾处理、雨水泵站等城市基础设施更加完善，城市精细化管理水平进一步提高。着力提升县域形象。加快县域经济发展，继续推进县城建设。大力开展“清洁家园·美丽乡村”活动，深入推进小城镇综合整治提升和乡村清洁工程，高标准建成15个示范乡镇、30个示范村和3条示范带，新改建农村公路850公里，90%以上乡镇基础设施和公共服务体系进一步完善，县域承载力继续提高。

　　（六）惠民生、促共享，社会大局和谐稳定。投入民生资金188.9亿元，增长20.9%，十项民生工程46项为民实事圆满完成。扎实推进精准扶贫。开展扶贫对象建档立卡和干部驻村帮扶工作，292个贫困村第一书记到岗就职；完成40个贫困村整村推进扶贫开发，3.6万农村贫困人口稳定脱贫。千方百计扩大就业。全市城镇新增就业7.2万人，新增农村劳动力转移就业3.7万人，城镇登记失业率控制在3.06%以内，始终保持城镇“零就业家庭”动态归零。继续实施全民技能振兴工程，完成各类职业技能培训13.5万人次。提高社会保障水平。城乡居民和企业退休人员基本养老金、城乡低保、农村五保、困难归侨侨眷补助等，全部按政策提高到位。社会养老服务体系进一步完善，新增养老机构8家，新增农村幸福养老院64家。做好关爱农村留守儿童、留守妇女、留守老人工作。残疾人、慈善、红十字事业健康发展。实施保障性安居工程。开工建设保障性住房和棚户区改造住房1.4万套，基本建成7467套；改造农村危房1.1万户。提高城乡教育水平。深入实施第二期学前教育三年行动计划，新建、改建幼儿园57所。全面改善农村义务教育薄弱学校基本办学条件，累计改造校园校舍289所。扩充城镇义务教育资源，新建、改扩建中小学校16所。职业教育园区建设步伐加快，职业教育水平不断提升。提高医疗健康水平。城乡居民人均基本公共卫生服务经费、城镇居民基本医疗保险和新农合医疗保险财政补助标准进一步提高。基层医疗卫生条件不断加强，全市行政村实现标准化卫生室全覆盖。持续开展食品药品安全专项治理，我市被评为省食品安全优秀城市。实施文化体育惠民工程。举办各类文化惠民活动2.5万场。开封文化客厅建成开放。成功举办2015郑开国际马拉松赛、市九运会暨首届全民健身大会，新建401个农民体育健身工程，乡镇农民体育健身工程覆盖率达到100%。汴京公园完成升级改造并向市民免费开放。创新加强社会治理。开展“三查三保”活动，确保安全生产责任制落实。进一步完善依法逐级走访、诉访分离和积案化解工作机制，推行信访代理制和“一单式”工作法，依法打击处置非法集资，非访集访案件大幅下降，守住了不发生区域性、系统性风险的底线。完成“六五”普法宣传，深化人民调解、行政调解和司法调解。开展“古城亮剑”集中行动，严厉打击违法犯罪和暴恐活动，人民群众安全感和满意度明显上升。

　　一年来，我们坚持不懈加强政府自身建设，认真开展“三严三实”专题教育和“一学两争三优”活动，坚决治理庸政懒政，加大巡察和督查力度，强化行政监察和审计监督，干净干事成为党员干部的自觉行动。坚持依法行政，自觉接受人大依法监督、政协民主监督和社会监督，认真办理人大代表建议和政协提案，满意和基本满意率达95.2%。大力开展国防教育，推进军民融合发展，驻汴解放军和武警部队在支持开封经济建设、维护社会稳定等方面做出了重要贡献。民族、宗教、统计、外事、侨务、对台、档案、史志、地震、人防、气象、社科研究、对口援疆等工作都取得了新成绩。

　　各位代表!

　　2015年各项目标任务的胜利完成，标志着我市“十二五”圆满收官，开封发展站在了新的历史起点上。“十二五”期间，全市各项建设取得显著成绩，是发展质量最好、城乡面貌变化最大、人民群众得到实惠最多的五年，是全市上下顽强拼搏、团结奋斗，奋发有为、积极进取的五年，是我们靠苦干换来成就，凭实力赢得尊重的五年。

　　——这五年，综合实力大幅跃升。生产总值从927亿元增加到1600亿元，年均增长10.7%，连续五年超过全省平均水平。一般公共预算收入从37亿元连续迈上50亿元、100亿元两个台阶，达到108.3亿元，年均增长23.9%，是2010年的2.9倍。农村居民和城镇居民人均可支配收入分别是2010年的1.9倍和1.7倍。

　　——这五年，经济结构不断优化。农村经济稳步发展，工业实力持续增强，文化旅游业亮点纷呈，新业态、新模式不断涌现，现代产业体系初步形成，产业规模逐步壮大，三次产业结构由23∶43.2∶33.8调整到17.4∶41.6∶41，二三产业比重上升5.6个百分点。

　　——这五年，发展动力持续增强。政府机构、行政审批、商事登记等重要领域和关键环节改革不断深化，制约发展的体制机制障碍得到有效破除。对外开放和招商引资持续保持全省领先，实际利用省外资金1840亿元，年均增长14.5%，是“十一五”末的2.5倍，是引资规模最大、签约项目最多、合同金额最高、招商效果最好的时期。

　　——这五年，城乡面貌显著改善。新一轮行政区划调整基本完成，新型城镇化步伐进一步加快，城镇化率达到44%。城乡一体化示范区初具规模，基础设施逐步完善，开封西湖蓄水成景，CBD建设全面启动。城市创建、环境整治和精细化管理成效显著，棚户区、城中村改造扎实推进，城市和农村面貌明显改观。

　　——这五年，社会文明程度全面提升。各项社会事业蓬勃发展，覆盖城乡居民的基本公共服务体系逐步完善。依法治市全面推进，民主法制建设更加健全。公民道德素质持续提高。生态环境不断改善。安全生产和信访形势继续保持平稳态势。平安开封建设取得显著成效，社会大局和谐稳定。

　　各位代表！

　　过去五年的成绩，是在经济形势异常严峻复杂、外部竞争异常激烈的条件下取得的，饱含着省委、省政府对开封的亲切关怀，体现着广大党员干部的责任与担当，凝聚着全市人民群众的智慧和汗水，这是市委正确领导、科学决策的结果，是市人大、市政协有效监督、大力支持的结果，是全市人民同心协力、团结奋斗的结果。在此，我代表市人民政府，向工作在全市各条战线上的工人、农民、知识分子、干部，向驻汴解放军指战员、武警官兵和公安民警，向各民主党派、工商联及社会各界人士致以崇高敬意！向所有关心、支持开封发展的同志们、朋友们表示衷心感谢！

　　各位代表！

　　回首“十二五”，我们遇到的困难比预想的大，取得的成绩比预期的好，发展的历程非同寻常，取得的经验弥足珍贵。五年来，我们始终坚持遵循规律、加快发展的战略定力。坚持发展是第一要务，抓牢发展不动摇，从“持续求快” 到“好中求快”，聚焦发展，精准施策，不懈怠、不停步、不退缩，以实实在在的成绩，赢得了发展上的主动权。五年来，我们始终坚持干事创业、勇于担当的工作激情。坚持一切从实际出发，不回避矛盾，不推脱责任，始终保持工作热情、创业激情，集中精力抓大事、谋要事、解难事，谋一件、干一件、成一件，一步一个脚印，一点一滴落实。五年来，我们始终坚持抢抓机遇、迎难而上的坚定自信。面对机遇不等待、不犹豫，牢记使命，务实奋进，坚持靠深化改革破解难题，靠扩大开放增强实力，一年一个台阶，不断焕发新活力，展现新形象。五年来，我们始终坚持统筹兼顾、协调推进的发展理念。统筹城乡发展，统筹老城新区，统筹产业布局，统筹改革开放，谋划建成一批事关经济社会发展全局的重大项目，办成一批既利当前又管长远的大事要事，经济建设、政治建设、文化建设、社会建设和生态文明建设协调推进。五年来，我们始终坚持改善民生、共享发展的根本宗旨。主动回应群众关切，连续实施十项民生工程，量力而行、尽力而为，一年接着一年干，最大限度谋福祉。

　　在肯定成绩的同时，我们也清醒地看到，我市经济社会发展中还存在不少矛盾和问题：经济实力依然偏弱，总量小、底子薄、外向度低的基本市情尚未根本改变。产业结构亟待优化，一产占比偏高，二产实力不强，现代服务业发展不够充分。创新驱动能力依然偏弱，新旧动力转换艰难，新增长点支撑能力不强，新业态、新模式仍需加大培育力度。资金、土地、环境等要素制约依然突出，体制机制障碍仍然较多。政府职能转变、简政放权还不到位，改革、发展和稳定任务十分艰巨。涉及人民群众切身利益的一些热点、难点问题还未彻底解决到位。安全生产仍有薄弱环节。对此，我们将高度重视，认真解决，努力让人民满意。

　　二、“十三五”发展的指导思想和目标任务

　　“十三五”时期是我市全面建成小康社会的决胜时期，是引领经济发展新常态、推进结构性改革的攻坚时期，也是拓展发展新空间、培育发展新动能的关键时期，更是力争经济实力进入全省第一方阵、建设河南省新兴副中心城市的黄金机遇期。综合判断，“十三五”时期，开封发展大有作为、大有可为，但也面临着矛盾叠加、风险隐患增多的严峻挑战。我国经济进入新常态，长期向好的基本面没有改变，但结构性产能过剩严重，中央提出“三去一降一补”五大任务，大力推进供给侧结构性改革，将全面启动市场需求，激发经济发展新动力。新一轮科技革命和产业变革蓬勃兴起，新技术新产品新成果转化带来新的增量，“双+”“双创”不断催生发展新活力。中央将出台中部崛起新十年计划，省委、省政府强力推进中原经济区和郑州航空港经济综合实验区建设，着力构建“一极三圈八轴带”发展格局，大力支持开封建设河南省新兴副中心城市，打造郑汴一体化升级版，必将释放新的发展潜力。经过多年的积累和探索，我市发展思路清晰坚定，综合实力不断增强，发展基础更加坚实，并形成了一套行之有效的体制机制，全市上下精诚团结、信心倍增，干事创业热情高涨，晋位升级、跨越发展的正能量正在汇聚。我们必须凝聚全市人民的智慧和力量，务实拼搏，砥砺前行，向全面建成小康社会目标奋勇前进。

　　今后五年，我市经济社会发展的指导思想是：高举中国特色社会主义伟大旗帜，全面贯彻党的十八大和十八届三中、四中、五中全会精神，以马克思列宁主义、毛泽东思想、邓小平理论、“三个代表”重要思想、科学发展观为指导，深入贯彻习近平总书记系列重要讲话精神，按照“五位一体”总体布局和“四个全面”战略布局，坚持发展是第一要务，牢固树立和贯彻落实创新、协调、绿色、开放、共享发展理念，围绕建设河南省新兴副中心城市总目标，强化新型工业化城市和国际文化旅游名城定位，抢抓“一带一路”战略、中原经济区与郑汴一体化发展、郑州航空港经济综合实验区建设重大机遇，着力优化经济结构，着力转换发展动力，着力厚植发展优势，着力保障和改善民生，建设实力开封、文化开封、美丽开封、幸福开封，力争经济实力进入全省第一方阵，确保如期全面建成小康社会。

　　今后五年，我市经济社会发展的主要目标是：建设实力开封。生产总值年均增长9%左右，一般公共预算收入年均增长10%左右，经济增长速度保持全省领先水平。城镇居民和农村居民人均可支配收入与经济增长同步，分别达到3.7万元和超过1.8万元。三次产业结构调整为12∶43∶45。城镇化率达到56%。建设文化开封。公共文化基础设施建设全面达到国家规定标准，覆盖城乡的公共文化服务体系基本健全。宋都古城文化产业园区特色彰显，国际文化旅游名城影响力不断增强。“文化+”行动取得显著成效，文化产业增加值占生产总值的比重达到7%，文化发展实力居全省前列。建设美丽开封。资源节约型和环境友好型城市建设取得新成效，主要污染物排放总量明显减少，大气和水质量达标率明显提高，生态安全屏障基本形成，林木覆盖率达到23%，城市建成区绿地率达到40%，自然生态人居环境明显改善。建设幸福开封。每年投入民生资金占公共预算支出比例不低于70%，社会事业全面进步，社会保障日趋完善，社会养老服务体系加快建立，居民生活质量、健康水平、居住条件明显提升，人民群众安全感、幸福感、获得感持续增强。

　　今后五年，我市经济社会发展的战略导向是：始终贯彻“五大理念”：把创新作为引领发展的第一动力，把协调作为持续健康发展的内在要求，把绿色作为永续发展的必要条件，把开放作为繁荣发展的必由之路，把共享作为加快发展的本质要求。实施重点战略举措：继续实施工业强市战略，推动工业经济提质增效；着力推进服务业发展，建设国际文化旅游名城；建设“四带三区”，打造郑汴一体化升级版；突出“两核六组团”，促进城乡协调发展；推进“双+”“双创”，培植发展新优势；加快对外开放步伐，构建改革开放新平台。

　　各位代表！

　　《开封市国民经济和社会发展第十三个五年规划纲要（草案）》及说明已印发各位代表，请与《政府工作报告》一并审议。

　　我们坚信，有省委、省政府和市委的坚强领导，有市人大、市政协的鼎力支持，有550万开封人民的奋勇拼搏，我市“十三五”发展蓝图一定能够如期实现！“四个开封”、河南省新兴副中心城市建设一定能够再奏凯歌、再谱新篇！

　　三、切实做好2016年工作，努力实现“十三五”良好开局

　　2016年是全面建成小康社会决胜阶段的开局之年，也是推进结构性改革的攻坚之年，做好今年各项工作，意义重大。

　　2016年我市经济社会发展主要预期目标是：生产总值增长9%左右，一般公共预算收入增长10%左右，规模以上工业增加值增长10%，全社会固定资产投资增长15%，社会消费品零售总额增长12%，进出口总值增长7%，实际到位省外资金增长10%，城镇新增就业5.5万人以上，常住人口城镇化率提高1.5个百分点左右，城乡居民收入增长与经济增长基本同步，居民消费价格涨幅3%左右，节能减排、环保等约束性指标完成省下任务，发展质量和效益明显提高，结构性改革取得实质性进展。

　　完成今年目标任务，必须做到“三个统一”，抓住“五个重点”，即：把认识和行动统一到中央对经济工作的准确判断上来，做到方向明；把责任和力量统一到省委、省政府对开封的定位和厚望上来，做到发力准；把目标和举措统一到我市“十三五”规划的总体部署上来，做到措施实。围绕“经济增长点”抓培育，围绕“精准发力点”扩增量，围绕“加快发展关键点”上项目，围绕“重要支撑点”强保障，围绕“共享发展落脚点”惠民生。重点抓好以下十个方面的工作：

　　（一）培育新的经济增长点。启动“开港经济带”建设，着力培育战略性新兴产业和现代物流业。紧密对接郑州航空港经济综合实验区，坚持高端谋划，高标准建设，确保成功申报国家产城融合示范区。加快开港大道建设，确保2017年7月竣工通车。有序展开产业布局，着力引进高附加值、高科技含量企业，重点发展高端制造业和现代物流业，加快已布局项目建设。以开港经济区为依托，积极申报保税物流中心。推进“示范区CBD”建设，着力培育现代服务业。完善基础设施和商业配套服务体系，确保5月底中意湖蓄水，年底前博物馆、规划馆、美术馆建成开放，在建的CBD商务楼宇群力争交付使用。依托已获批的国家级电子商务示范基地，新培育50家电子商务企业。加快绿地中部创客天地项目建设，搭建创客集聚平台、研发支撑平台，再建一批众创空间，打造“双创”活力地带。大力培育现代金融新业态，做大做强金融业。加快大河印象文化城等项目建设，积极发展文化创意产业。推进“宋都古城文化产业园区”建设，全面提升文化旅游业。加快宋都文化商务区、城摞城新郑门遗址、地下城墙展示馆、小宋城二期、艮岳宋代园林博物馆等项目建设。建成中原明珠文化旅游产业园、建业东京梦华半亩园、双龙巷历史街区改造等项目，启动清明上河园三期、建业泰和府、非遗创意园等项目建设，启动大相国寺周边综合改造，完成马道街街景整治。设立文化旅游产业发展基金，加强对文化旅游企业引导扶持。办好清明文化节、菊花文化节、收藏文化论坛和国际茶文化研讨会。以创建全国旅游标准化示范城市为契机，加快旅游集散中心建设，完善旅游基础设施，加大开封旅游整体宣传推介，促进文化旅游业升级发展。启动“东部新城”建设，着力培育新能源新材料产业。高标准编制总体规划，启动以祥符区城区为核心的东部新城建设，谋划好新城发展和产业布局，力争核心城区“一年一个样、三年大变样、五年成规模”。做好祥符区与老城区基础设施对接共享，贯通华夏大道至祥符区，逐步完善配套基础设施。做大做强主导产业，重点培育新能源新材料、高端化工、精细化工产业，夯实东部新城产业基础。打造“郑汴一体化升级版”亮点，着力培育产学研和大健康产业。启动郑汴产学研结合示范带、沿黄生态带规划编制和建设工作。完善职业教育园区基础设施，开工建设省外贸学校、军威科技职业学院、化工职业学院，力争再筹建一所工科类学校。加强职业院校与企业合作，推动各类科研成果和实用技术转化应用，布局延伸产学研产业。积极发展大健康产业，确保中心医院、体育中心等项目开工建设，做大做强生物医药和高端医疗器械制造等产业，积极培育健康养老、养生休闲、体育消费等新兴业态。以实施“双+”“双创”为契机，催生新业态新模式。落实好“双+”行动计划、“双创”工作意见，用好“文化+”发展资金，开展“文化+”十大专项行动。实施“513”工程，即实施文化旅游、商贸流通、健康养老、房地产、“文化＋”5大类超过100个服务业重点项目，力争全年投资达到300亿元。创新“互联网+”新模式，着手实施数据整合工程，提升城市智能化水平。继续实施科技企业孵化器发展三年行动计划，推广创客空间、创新工场等新型孵化模式，打造创新创业生态全链条。

　　（二）促进工业经济提质增效。积极推进供给侧改革。出台扶持工业经济发展的政策措施，稳定优质存量，持续扩大有效增量。深入开展服务企业活动，研究制定“降本增效”跟进措施，切实减轻企业负担。对长期亏损、资不抵债、停产半停产的“僵尸企业”实行兼并重组，妥善处置。支持研发新产品。用好5000万元工业转型升级专项基金，引导企业加快新技术转化和新产品研发。加快奇瑞汽车SUV新车型、通海流体核电级专用阀门等新产品研发进度。实施质量兴市战略，实现省长质量奖“零突破”。开展“文化+工业”专项行动，加快汴绣文化研发基地等项目建设，推动新型工业化与“文商旅”模式融合发展。加快转化新技术。支持“双50”企业建设高水平技术研发中心，确保国家空分设备质量监督检验中心建成投用。推进开封炭素、开封仪表等企业技术升级改造，加快新技术成果转化。大力实施新项目。严格按时间节点推进重点项目“321”工程，即实施300个以上重点项目，总投资超过2000亿元，全年完成投资超过1000亿元，着力抓好32个省级A类重点项目。实施“7131”工业项目工程，围绕7大类产业实施150个以上工业项目，力争完成工业项目投资300亿元，带动全市工业投资增长10%以上。重点推动易成新能负极新材料、优德医疗设备等一批重大工业项目建设。完善产业体系。调整产业发展重点，依托产业集聚区和服务业“两区”，发展壮大装备制造及汽车和零部件、农产品加工、化工、纺织服饰等4个传统产业集群，积极培育新能源新材料、电子信息、生物医药等3个新兴产业集群，着力推动文化旅游、商贸物流、健康养老3个服务业产业集群发展，构建“4+3+3”产业体系，加快培育百、千亿级优势产业集群。突出整产业链发展，加快产业集聚区建设，努力形成一批有特色、有规模、具有核心竞争力的特色专业园。

　　（三）推动服务业转型升级。壮大服务业企业规模。落实好服务业发展指导意见，发挥3000万元服务业引导基金作用，推动商贸流通、特色餐饮等传统服务业转型升级。大力发展现代金融、教育培训、科技服务等高成长性服务业。实施消费产品升级工程，加快培育百家名店、百家老字号。鼓励服务业企业抱团发展、集聚发展，力争入规入库服务业企业300家。加快中心商务区、特色商业区建设。把发展重心转到促集聚、育特色、提服务、强融合上，打造10个以上主营业务收入超亿元的服务业“两区”和特色市场。加快开封国际农业会展中心等42个投资亿元以上“两区”重点项目建设，力争全市服务业“两区”固定资产投资完成100亿元，主营业务收入增长15%以上。培育新的消费增长点。启动实施新兴热点消费促进工程，研究刺激消费政策措施，搞活以互联网为载体、线上线下互动的新兴消费，促进养老家政健康消费，扩大教育文化体育消费，持续提升农村消费能力。大力发展电子商务，全市新增电商企业、电商商户1000家以上。促进房地产业健康发展。落实好促进房地产业健康发展各项政策措施，完善进城农民购房激励政策，引导房地产企业顺应市场规律，合理确定商品房价格。统筹保障性住房和商品房建设，探索施行收购存量商品住房用于保障房政策，打通商品房、安置房、保障房转换渠道，加快消化房地产库存。

　　（四）统筹推进农业农村工作。打好扶贫攻坚战。按照扶持对象精准、项目安排精准、资金使用精准、措施到户精准、因村派人精准、脱贫成效精准的要求，“转、扶、搬、保、救”五措并举，实施扶贫攻坚“五个一批”工程，完成扶贫开发整村推进村50个，再实现3.5万农村贫困人口稳定脱贫。稳定提高粮食产量。实施藏粮于地、藏粮于技战略，推进高标准粮田建设，确保粮食总产实现“十三连增”。加快农业科技创新及成果转化，提高农业综合机械化率，持续推进气象防灾减灾和为农服务体系现代化建设。持续抓好灌区续建配套与节水改造、中小河流治理、病险水闸加固等水利工程建设，力争每县启动建设一处引黄调蓄工程，新增改善有效灌溉面积26万亩。打通农产品全产业链。树立大农业、大食物观念，推进农村一二三产业融合发展。围绕生猪、肉鸡、肉牛、大蒜等优质农产品，重点打通9条农产品全产业链，支持牧原、天丰、大用、包公食品等农业产业化龙头企业发展壮大，新培育现代农业产业化集群3个，力争全市农产品加工业增加值超过400亿元。探索多种交叉融合方式，培育农村电商、农产品定制等“互联网+”新业态。实施都市生态农业发展工程，选择6个有条件的乡镇，积极发展休闲观光农业，拓宽农民就业增收空间。持续深化农村改革。基本完成农村土地承包经营权确权登记颁证工作，全面启动农村集体建设用地使用权确权登记发证工作，出台引导农村土地经营权有序流转实施方案。落实农业“三项补贴”改革，发展种粮大户、家庭农场等新型农业经营主体，健全农业公共服务和社会化服务体系，发挥农业适度规模经营在结构性改革中的引领作用。

　　（五）促进城乡协调发展。协调“两核六组团”发展。“两核”定位：老城区以城墙内13平方公里为核心，以宋都古城文化产业园区为载体，以宋文化为主题、文商旅融合为重点，努力建设成为全城一景、宋韵彰显的国际文化旅游名城目的地。示范区以CBD为核心，以开港经济带、郑汴产学研结合示范带、沿黄生态带为载体，以发展现代服务业、装备制造及汽车和零部件产业为重点，实施二次创业，开展第二个五年攻坚，全力打造郑汴一体化升级版，努力建设成为河南省新兴副中心城市的核心增长极。“六组团”定位：东部新城组团以祥符区城区为核心，祥符区、顺河回族区、禹王台区多组团协调发展，以黄龙、汴东、精细化工产业集聚区为载体，以发展新能源新材料、精细化工、高端化工为重点，努力建设成为区域协调发展的东部新城。朱仙镇组团以朱仙镇古镇为核心，依托开港经济带重要节点优势，以朱仙镇文化旅游发展试验区为载体，以文化体验、休闲度假、健康养老、科技教育为产业重点，努力建设成为享誉全国的历史文化古镇。尉氏县组团以建设全省经济综合实力“20强县”为总目标，强化郑汴一体化南部经济重心、中原经济区产城融合样板、郑州航空港经济综合实验区东部创新高地“三个定位”，以纺织服饰、健康医疗、现代家居为产业重点，努力建设成为全市县域经济发展的“排头兵”。杞县组团以打通南部经济带，开通郑民高速二期为契机，主动对接市区、联接港区，在持续壮大新能源新材料产业的同时，打造“粮经饲”统筹、“种养加”一体全产业链条，努力建设成为一二三产融合的示范区。通许县组团以打通南部经济带为契机，依托交通区位优势，主动对接市区、联接港区，深挖产业潜力，瞄准高端、高质、高新，以中低速电动汽车、电动特种车转型升级为重点，努力建设成为制造业转型升级的先行区。兰考县组团充分发挥省直管优势，建设地区副中心城市。加快中心城市建设。尊重城市发展规律，坚持人民城市为人民，走出一条具有开封特色的城市发展道路。高度重视古城保护和历史文脉延续，完成城市风格规划设计，加强对建筑设计、城市风貌的约束和指导。继续做好“两改一建”工作，实施24个棚户区、城中村改造项目，征收房屋7798户、183.6万平方米，解决33个棚户区、城中村改造项目及73个保障性住房项目遗留问题。开展精准化房屋征收，该征收的坚决征收到位，不留后遗症，能保护的坚决保护，避免大拆大建。巩固国家卫生城市、园林城市创建成果，以创建全国文明城市为载体，提高城市精细化管理水平，加强市民文明教育，市民素质高一分，城市形象美十分，让每一位开封市民都成为城市文明形象代言人。进一步改善城乡面貌。启动提升县级城市管理水平三年行动计划。继续开展“清洁家园·美丽乡村”和小城镇综合整治提升工程，推进原“133工程”连点、串线、扩面，实施新“133工程”，再建设100个美丽乡村、30个示范乡镇和30个活力社区，着力改善农村人居环境。加强指导，分类推进，妥善解决87个新型农村社区遗留问题。加快农业转移人口市民化。按照“一基本两牵动三保障”总体要求，推进以满足新市民为出发点的住房制度改革，推动户籍制度改革和居住证制度“双落地”。加强城乡社会保障制度衔接，维护好进城农民土地承包权、宅基地使用权、集体收益分配权。

　　（六）完善以“八网”为重点的基础设施建设。完善路网。重点完善城铁、高铁、高速路、快速路、干支线路、城市道路和公交线路，提高道路通行能力。启动郑开城际铁路延长线项目建设；建成郑开城际铁路宋城站、郑徐高铁开封北站广场及配套工程；确保郑民高速二期建成通车，继续推进机西高速二期（开封段）建设，完成郑民高速南苑站扩建工程，开工建设连霍高速开港大道出入口，做好连霍高速开柳路出入口前期工作；启动开尉路、开杞路改建和祥港大道建设工程；完成“农村公路三年行动计划”。全面建成2015年已开工道路。以打通断头路为重点，新开工建设孝严寺街（内顺城路-孝严寺桥）、夷山大街土城段（万胜路-电视塔南路）、内顺城路（龙亭北路西口-万岁山南口）、龙亭西路北延（万岁山南口-西关北街-东京大道）、东外环路北延（东京大道-复兴大道）、魏都路东延（五一路-中山路）等6条道路。继续大力发展公共交通，调整优化15条公交线路，加快宋城客运枢纽站、小李庄公交首末站等公交场站建设。完善水网。重点完善水系、引黄水利工程、雨污水管网、供水管网。开工建设涧水河工程，改扩建水系一期，启动建设开封西湖二期、碧水河、秀溪河、王庵水库、运粮河调蓄工程、引黄入杞工程。做好龙亭湖、包公湖、黑池清淤，治理黄汴河（东京大道孙李唐-滨河路皮革厂桥）、东护城河（东京大道仁和屯-滨河路皮革厂桥）、东干渠（建设路-化肥河）、化肥河、惠济河（侯庄桥-文庄桥）、北郊沟（发源地-东京大道仁和屯）6条黑臭水体。启动老城区第三污水处理厂、示范区第二污水处理厂建设，新建东京大道西外环路雨水泵站及东外环路、夷山大街北延污水管网。积极争取南水北调工程入汴，增加新的城市水源。完善热网。重点加快热源和配套管网建设，积极推进东区供热，市区新增供热面积350万平方米，新增供热家庭1.8万户，比2015年翻一番。完善气网。重点加强天然气配套管网建设，提高燃气应急保障能力。新建燃气管线77公里，市区燃气气化率达到90%以上。完善电网。加快22个110千伏及以上输变电工程建设，新建35千伏及以下供电线路600千米，提升全市总供电能力到354万千瓦。完善光网。全面推进宽带光纤化，城乡无线宽带速率提高到150M。新建50万光纤宽带端口，家庭宽带用户宽带接入能力达到20M以上。完善绿网。重点加强生态廊道、湿地、公园绿地及道路河渠两侧绿化，提升生态廊道绿化水平。实施惠济河湿地、马家河湿地综合保护工程，涵养“城市绿肺”。提升植物园绿化水平，开工建设环城墙公园六期（明伦街-汴京公园北围墙）、七期（地震台-万岁山），建设城市绿带。完善云网。积极整合政务云、企业云、旅游云、文化云等云端资源，加快智慧城市建设，建成大数据中心和智慧开封展示体验中心，启动应急指挥中心建设，力争再开工一批智慧项目。

　　（七）加强支撑保障体系建设。继续推进改革。深化行政体制改革，持续推进投资审批、收费清理、商事制度等改革。加快行政审批四级便民服务体系和标准化建设，建成30个“市民之窗”便民自助服务平台。深化国有企业改革，完善国有企业外派监事会制度，合理界定国有企业功能类别，实施分类改革、发展和监管。全面清理限制非公有制经济发展的不合理规定，促进非公有制经济持续快速健康发展。深化财税金融和投融资体制改革，推进消费税改革和资源税费改革，做好国税、地税征管体制综合改革。加快县域农信社组建为农商行，积极引进港中旅银行、郑州银行、中信银行等股份制银行，争取浦发银行档案中心、邮储银行办公中心、中原银行培训中心等后台服务中心落户开封。创新PPP项目收益和分配机制，完善公共产品价格形成机制。统筹抓好其它领域改革。实施创新驱动。激发企业、科研机构和高校、市场转化科技成果潜力。发挥“双+”、“双创”乘数效应，新培育高新技术企业5家、工程技术研究中心10家、市级重点实验室2~3家。加强新型研发机构、科技研发基地合作平台建设，新建众创空间、孵化器10家，培育科技型中小微企业100家。保护生态环境。深入实施蓝天、碧水、乡村清洁等工程，留住“碧水蓝天”。继续推进燃煤锅炉清洁能源改造或拆除，加大黄标车淘汰力度，加强城区运输车辆及施工工地扬尘污染治理。加大秸秆禁烧力度，做好重污染天气应急应对，遏制雾霾天气高发。继续实施水源地环境综合整治六大工程，强化地表和地下水污染防治，保障饮用水安全。继续实施城市清洁河流行动计划，强力推进城市排污治理。持续推进农业面源治理、土壤污染治理和林业生态市建设，力争创建4个省级生态乡镇和18个省级生态村。强化要素保障。坚持最严格的耕地保护制度，坚守耕地红线。更加注重土地挖潜，促进土地集约、节约利用。对批而未征、征而未供、供而未用的项目用地依法分类处理，达到收回条件的坚决予以收回，对因政府原因造成征收慢、供地慢的行为坚决查处。创新金融产品，扩大信贷投放，引导外资和民间资本采用PPP模式参与城市建设，用好已签约的300亿元城市发展基金。积极推进企业上市，实现7家企业上市融资。实施最严格的环境保护制度，建立健全覆盖所有固定污染源的企业排放许可、评价考核与责任追究制度，严厉打击环境违法行为，为发展留足环境容量。继续实施全民技能振兴工程，完成各类职业技能培训13.8万人次。加强市校合作，推进招才引智“2231”人才工程，提升人力资源保障能力。

　　（八）提升开放带动水平。深度融入“一带一路”。发挥“一带一路”城市旅游联盟作用，加强与国内外城市合作交流，携手推动更大范围、更高水平、更深层次的开放。鼓励企业走出去，支持空分、化工、农产品加工、空间结构等企业开拓海外市场。积极发展跨境电子商务。突出抓好开放招商。以十大产业集群和新技术、新业态、新模式招商为重点，以延链补链强链为方向，着力抓好具有引领性、突破性、方向性的重大项目引进。用好招商引资“六项机制”，强化三个招商链条，紧盯重点区域精准招商、联合招商、整体招商，鼓励现有企业增资扩股实现零地招商，力争全年引进亿元以上项目130个以上，实际到位省外资金增长10%以上，实际利用外资增长9%以上，新批千万美元以上外资项目20个。强化跟踪服务，提高招商实效，2014年和2015年签约的318个项目开工率达到50%以上。建好用好招商平台。充分发挥四个招商基地作用。全力做好河南自贸区（开封片区）申报工作，争取在开封设立海关、商检、跨境电子商务基地等机构，推动上海自贸区“28+6”经验复制落地。

　　（九）持续保障和改善民生。继续实施十项民生工程，办好为民实事。推动更高质量就业。实施更加积极的创业就业政策，进一步健全公共就业服务体系，扎实开展公共就业服务活动，打造全方位、立体化的就业服务网络，确保城镇新增就业5.5万人以上，农村劳动力转移就业3万人。提升社会保障水平。全面实施全民参保登记计划，推动社会保障由制度全覆盖转向法定人员全覆盖，确保城镇职工、城乡居民养老金按时足额发放。统筹推进城乡社会救助体系建设，完善最低生活保障、特困人员供养、残疾人帮扶等兜底性制度。健全关爱“三留守”人员工作机制。积极发展残疾人事业和慈善事业。努力办好人民满意的教育。科学规划，合理布局，完成第二期学前教育三年行动计划，新建、改扩建幼儿园44所，扶持普惠性民办幼儿园40所。深入推进全面改善义务教育薄弱学校基本办学条件和扩充城镇义务教育资源五年规划，新建中小学校6所、改扩建12所，促进义务教育均衡发展。推动普通高中内涵特色多样化发展。加快各类职业教育发展水平。支持河南大学、黄河水利职业技术学院等高校发展，办好开封大学、开封文化艺术职业学院。完善助学资助体系，让更多贫困家庭孩子有机会接受更好教育。提高人民健康水平。强化基层卫生服务能力建设，推进公共卫生服务均等化。做好重大传染病防控和公共卫生事件应急处置。支持中医药事业发展。实施全面“两孩”政策，改革完善计划生育服务管理。广泛开展全民健身运动，建成城墙体育公园，新建240个农民体育工程、46个健身路径工程。提高体育竞技水平，办好2016中国郑开国际马拉松赛和中国（开封）客家国际龙舟邀请赛。扩大基本公共文化服务。继续实施文化惠民全覆盖工程，组织开展“舞台艺术送农民”、政府购买公益性演出、电影公益放映活动。加快推进公共文化基础设施建设，高标准建成市美术馆，启动市图书馆、群艺馆新馆前期工作。拓展公共文化服务场所免费开放功能，大力倡导全民阅读，丰富群众精神生活。加强非物质文化遗产的传承、保护和利用，积极推进开封城墙申报世界文化遗产。防范和化解各类风险。严格落实安全生产责任制，坚决防范和遏制重特大安全事故发生。完善食品药品安全监管体制机制，防止食品药品安全事件发生，积极创建全国食品安全示范城市。落实属地管理责任和监管部门责任，集中解决非法集资问题，最大限度维护群众利益、维护经济发展、维护社会稳定。完善社会治理。健全社会治安立体防控体系，完善反恐和打击犯罪机制，强化流动人口和特殊人群服务管理，落实社区矫正工作。做好信访维稳工作，更加注重依法化解社会矛盾。加强社区建设，推行政府购买社区社会服务试点。加强互联网舆论引导与管理，努力打造清朗网络空间。

　　继续加强国防动员、国防教育和国防后备力量建设，支持开封驻军、武警部队、预备役部队、民兵和人民防空建设，深入开展双拥共建，巩固军政军民团结，推动军民深度融合发展。支持工会、共青团、妇联等人民团体更好发挥桥梁纽带作用，注重发挥工商联等社会团体作用。依法管理民族宗教事务，大力发展民族经济，促进民族团结进步、宗教关系和谐。加强统计、督查工作。继续做好外事、侨务、港澳、对台、史志、地震、档案、社科研究等工作。

　　（十）打造人民满意的政府。打铁还需自身硬。今年我们将以更大的决心、更实的措施，持续推进法治政府、服务政府、廉洁政府、责任政府建设。坚持依法行政。牢固树立法治理念，运用法治思维和法治方式解决问题，按照法定权限和程序行使权力、履行职责。坚持依法科学民主立法，推进市容环境卫生、饮用水水源保护等立法。健全依法决策机制，完善公众参与、专家论证、风险评估、合法性审查等重大行政决策法定程序。强化依法治理，启动“七五”普法。推进服务型行政执法，促进严格规范公正文明执法。转变工作作风。持续践行“三严三实”，驰而不息纠正“四风”，健全岗位履职责任和激励机制，健全全面覆盖的督查机制，完善效能问责办法，解决“中梗阻”，让那些身在岗位不作为、拿着俸禄不干事、占着位置不担当、庸政懒政怠政的政府公务人员绝无立身之地。强化责任担当。保持昂扬向上、积极进取精神状态，面对矛盾困难敢于迎难而上，面对深层次难题敢于知难而进，做到守土有责、奋发有为，政府提出的规划目标、发展任务、民生实事、作出的承诺，做到事事有时间表，人人有任务书，步步有路线图，说了算，定了干，干到成。依法接受监督。强化行政权力制约和监督，加强和改善事前事中事后监管，全面推进政务公开。依法接受人大的法律监督和工作监督，认真落实市人大及其常委会的各项决议决定，坚持政府重大决策向人大报告制度。主动加强同人民政协的联系，积极支持市政协履行政治协商、民主监督和参政议政职能，认真办理人大代表建议和政协提案。保持清正廉洁。始终把纪律挺在前面，认真履行主体责任和“一岗双责”，严格落实党风廉政建设责任制，严格执行廉政准则和各项制度规定，强化行政监察和审计监督，坚决查处权力寻租、权钱交易和损害群众利益的行为，以零容忍态度惩治腐败，以反腐倡廉的实际成效保障发展、取信于民！

　　各位代表！

　　人民群众的期待就是我们努力的方向！人民群众的所需就是我们奋斗的目标！当前，开封的发展已经站在新的历史起点上，前景令人鼓舞，梦想催人奋进。让我们紧密团结在以习近平同志为总书记的党中央周围，高举中国特色社会主义伟大旗帜，在省委、省政府和市委的坚强领导下，紧紧依靠和团结带领全市人民，继续务实苦干，奋勇拼搏，努力把“十三五”规划的宏伟蓝图变为现实，让无限的希望和梦想变为现实，为加快建设实力开封、文化开封、美丽开封、幸福开封，加快建设河南省新兴副中心城市，全面建成小康社会而努力奋斗！

　　在华夏大道、开封西湖、建业铂尔曼酒店等项目顺利建成，商登高速、机西高速（开封段）建成通车，郑徐高铁开封北站、德豪光电科技产业园等项目扎实推进的同时，新谋划启动开港大道、郑开城际铁路延长线、涧水河、绿地中部创客天地、智慧城市三中心等一批重大项目。面向全国发布首批30个PPP项目，5个进入执行阶段。完善项目推进服务机制。实施重大项目领导分包、县区会商、联席会议等“六项机制”，健全“招商链、审批链、推进链、保障链”全链条服务，成立市委市政府督查局和市企业投诉中心，加大项目督导力度，狠抓项目跟踪推进。绘制“两图”，编织“两网”，基本实现市、县、乡（镇）、村（社区）四级审批联网，推行“一站式”政务服务，企业和群众办事更加便捷。

　　（四）促改革、扩开放，发展动力活力进一步释放。全力推进改革。深化行政体制改革，新一轮政府机构改革全面完成，市政府工作部门全部建立权力清单和责任清单；推行“三证合一”商事登记制度；全面取消非行政许可审批，行政审批事项由182项精简到129项。深化农村改革，334.7万亩农村土地承包经营权确权登记基本完成，宅基地和集体建设用地使用权确权登记发证有序推进。深化医疗卫生体制改革，实施基本药物零差率销售，县级公立医院综合改革全面启动。机关事业单位工资和养老保险制度、事业单位分类改革扎实推进，交通运输综合执法体制、党政机关公务用车制度改革顺利完成，国有企业、投融资、价格、食品药品监管、司法体制等改革取得新进展。持续扩大开放。建立四个招商基地，完善招商引资“六项机制”，成功举办上海产业合作推介会、京津区域经济技术合作洽谈会等重大招商活动，全市签约项目168个，签约资金1183亿元；实际到位省外资金450亿元，增长10.7%；实际利用外资5.6亿美元，增长20.6%；进出口总值增长3.1%。成立市口岸办，有序推进河南自贸区（开封片区）申报工作。全面启动城市旅游联盟合作，加强与友好城市交流，对外开放迈出坚实步伐。

　　（五）优环境、重协调，城市品位显著提升。加强生态环境保护。扎实推进节能减排，化学需氧量、氨氮、二氧化硫排放量完成省下目标。严守生态红线，实施蓝天、碧水等工程，全市空气质量优良天数222天，居全省第2位；地表水水质平均达标率93.8%，继续位居全省前列。加强林业生态市建设，完成造林9.4万亩。改善新老城区面貌。投资30亿元，加快示范区基础设施建设，中意湖、“三站三馆两中心”等CBD周边项目加快推进，开封西湖形成靓丽景观，示范区形象显著提升。加快老城区改造步伐，完成棚户区、城中村改造164.8万平方米，拓宽改造内环路、内顺城路、魏都路等一批城市道路，环城墙公园、御河水系等城市景观带建设扎实推进，停车场、污水及垃圾处理、雨水泵站等城市基础设施更加完善，城市精细化管理水平进一步提高。着力提升县域形象。加快县域经济发展，继续推进县城建设。大力开展“清洁家园·美丽乡村”活动，深入推进小城镇综合整治提升和乡村清洁工程，高标准建成15个示范乡镇、30个示范村和3条示范带，新改建农村公路850公里，90%以上乡镇基础设施和公共服务体系进一步完善，县域承载力继续提高。

　　（六）惠民生、促共享，社会大局和谐稳定。投入民生资金188.9亿元，增长20.9%，十项民生工程46项为民实事圆满完成。扎实推进精准扶贫。开展扶贫对象建档立卡和干部驻村帮扶工作，292个贫困村第一书记到岗就职；完成40个贫困村整村推进扶贫开发，3.6万农村贫困人口稳定脱贫。千方百计扩大就业。全市城镇新增就业7.2万人，新增农村劳动力转移就业3.7万人，城镇登记失业率控制在3.06%以内，始终保持城镇“零就业家庭”动态归零。继续实施全民技能振兴工程，完成各类职业技能培训13.5万人次。提高社会保障水平。城乡居民和企业退休人员基本养老金、城乡低保、农村五保、困难归侨侨眷补助等，全部按政策提高到位。社会养老服务体系进一步完善，新增养老机构8家，新增农村幸福养老院64家。做好关爱农村留守儿童、留守妇女、留守老人工作。残疾人、慈善、红十字事业健康发展。实施保障性安居工程。开工建设保障性住房和棚户区改造住房1.4万套，基本建成7467套；改造农村危房1.1万户。提高城乡教育水平。深入实施第二期学前教育三年行动计划，新建、改建幼儿园57所。全面改善农村义务教育薄弱学校基本办学条件，累计改造校园校舍289所。扩充城镇义务教育资源，新建、改扩建中小学校16所。职业教育园区建设步伐加快，职业教育水平不断提升。提高医疗健康水平。城乡居民人均基本公共卫生服务经费、城镇居民基本医疗保险和新农合医疗保险财政补助标准进一步提高。基层医疗卫生条件不断加强，全市行政村实现标准化卫生室全覆盖。持续开展食品药品安全专项治理，我市被评为省食品安全优秀城市。实施文化体育惠民工程。举办各类文化惠民活动2.5万场。开封文化客厅建成开放。成功举办2015郑开国际马拉松赛、市九运会暨首届全民健身大会，新建401个农民体育健身工程，乡镇农民体育健身工程覆盖率达到100%。汴京公园完成升级改造并向市民免费开放。创新加强社会治理。开展“三查三保”活动，确保安全生产责任制落实。进一步完善依法逐级走访、诉访分离和积案化解工作机制，推行信访代理制和“一单式”工作法，依法打击处置非法集资，非访集访案件大幅下降，守住了不发生区域性、系统性风险的底线。完成“六五”普法宣传，深化人民调解、行政调解和司法调解。开展“古城亮剑”集中行动，严厉打击违法犯罪和暴恐活动，人民群众安全感和满意度明显上升。

　　一年来，我们坚持不懈加强政府自身建设，认真开展“三严三实”专题教育和“一学两争三优”活动，坚决治理庸政懒政，加大巡察和督查力度，强化行政监察和审计监督，干净干事成为党员干部的自觉行动。坚持依法行政，自觉接受人大依法监督、政协民主监督和社会监督，认真办理人大代表建议和政协提案，满意和基本满意率达95.2%。大力开展国防教育，推进军民融合发展，驻汴解放军和武警部队在支持开封经济建设、维护社会稳定等方面做出了重要贡献。民族、宗教、统计、外事、侨务、对台、档案、史志、地震、人防、气象、社科研究、对口援疆等工作都取得了新成绩。

　　各位代表!

　　2015年各项目标任务的胜利完成，标志着我市“十二五”圆满收官，开封发展站在了新的历史起点上。“十二五”期间，全市各项建设取得显著成绩，是发展质量最好、城乡面貌变化最大、人民群众得到实惠最多的五年，是全市上下顽强拼搏、团结奋斗，奋发有为、积极进取的五年，是我们靠苦干换来成就，凭实力赢得尊重的五年。

　　——这五年，综合实力大幅跃升。生产总值从927亿元增加到1600亿元，年均增长10.7%，连续五年超过全省平均水平。一般公共预算收入从37亿元连续迈上50亿元、100亿元两个台阶，达到108.3亿元，年均增长23.9%，是2010年的2.9倍。农村居民和城镇居民人均可支配收入分别是2010年的1.9倍和1.7倍。

　　——这五年，经济结构不断优化。农村经济稳步发展，工业实力持续增强，文化旅游业亮点纷呈，新业态、新模式不断涌现，现代产业体系初步形成，产业规模逐步壮大，三次产业结构由23∶43.2∶33.8调整到17.4∶41.6∶41，二三产业比重上升5.6个百分点。

　　——这五年，发展动力持续增强。政府机构、行政审批、商事登记等重要领域和关键环节改革不断深化，制约发展的体制机制障碍得到有效破除。对外开放和招商引资持续保持全省领先，实际利用省外资金1840亿元，年均增长14.5%，是“十一五”末的2.5倍，是引资规模最大、签约项目最多、合同金额最高、招商效果最好的时期。

　　——这五年，城乡面貌显著改善。新一轮行政区划调整基本完成，新型城镇化步伐进一步加快，城镇化率达到44%。城乡一体化示范区初具规模，基础设施逐步完善，开封西湖蓄水成景，CBD建设全面启动。城市创建、环境整治和精细化管理成效显著，棚户区、城中村改造扎实推进，城市和农村面貌明显改观。

　　——这五年，社会文明程度全面提升。各项社会事业蓬勃发展，覆盖城乡居民的基本公共服务体系逐步完善。依法治市全面推进，民主法制建设更加健全。公民道德素质持续提高。生态环境不断改善。安全生产和信访形势继续保持平稳态势。平安开封建设取得显著成效，社会大局和谐稳定。

　　各位代表！

　　过去五年的成绩，是在经济形势异常严峻复杂、外部竞争异常激烈的条件下取得的，饱含着省委、省政府对开封的亲切关怀，体现着广大党员干部的责任与担当，凝聚着全市人民群众的智慧和汗水，这是市委正确领导、科学决策的结果，是市人大、市政协有效监督、大力支持的结果，是全市人民同心协力、团结奋斗的结果。在此，我代表市人民政府，向工作在全市各条战线上的工人、农民、知识分子、干部，向驻汴解放军指战员、武警官兵和公安民警，向各民主党派、工商联及社会各界人士致以崇高敬意！向所有关心、支持开封发展的同志们、朋友们表示衷心感谢！

　　各位代表！

　　回首“十二五”，我们遇到的困难比预想的大，取得的成绩比预期的好，发展的历程非同寻常，取得的经验弥足珍贵。五年来，我们始终坚持遵循规律、加快发展的战略定力。坚持发展是第一要务，抓牢发展不动摇，从“持续求快” 到“好中求快”，聚焦发展，精准施策，不懈怠、不停步、不退缩，以实实在在的成绩，赢得了发展上的主动权。五年来，我们始终坚持干事创业、勇于担当的工作激情。坚持一切从实际出发，不回避矛盾，不推脱责任，始终保持工作热情、创业激情，集中精力抓大事、谋要事、解难事，谋一件、干一件、成一件，一步一个脚印，一点一滴落实。五年来，我们始终坚持抢抓机遇、迎难而上的坚定自信。面对机遇不等待、不犹豫，牢记使命，务实奋进，坚持靠深化改革破解难题，靠扩大开放增强实力，一年一个台阶，不断焕发新活力，展现新形象。五年来，我们始终坚持统筹兼顾、协调推进的发展理念。统筹城乡发展，统筹老城新区，统筹产业布局，统筹改革开放，谋划建成一批事关经济社会发展全局的重大项目，办成一批既利当前又管长远的大事要事，经济建设、政治建设、文化建设、社会建设和生态文明建设协调推进。五年来，我们始终坚持改善民生、共享发展的根本宗旨。主动回应群众关切，连续实施十项民生工程，量力而行、尽力而为，一年接着一年干，最大限度谋福祉。

　　在肯定成绩的同时，我们也清醒地看到，我市经济社会发展中还存在不少矛盾和问题：经济实力依然偏弱，总量小、底子薄、外向度低的基本市情尚未根本改变。产业结构亟待优化，一产占比偏高，二产实力不强，现代服务业发展不够充分。创新驱动能力依然偏弱，新旧动力转换艰难，新增长点支撑能力不强，新业态、新模式仍需加大培育力度。资金、土地、环境等要素制约依然突出，体制机制障碍仍然较多。政府职能转变、简政放权还不到位，改革、发展和稳定任务十分艰巨。涉及人民群众切身利益的一些热点、难点问题还未彻底解决到位。安全生产仍有薄弱环节。对此，我们将高度重视，认真解决，努力让人民满意。

　　二、“十三五”发展的指导思想和目标任务

　　“十三五”时期是我市全面建成小康社会的决胜时期，是引领经济发展新常态、推进结构性改革的攻坚时期，也是拓展发展新空间、培育发展新动能的关键时期，更是力争经济实力进入全省第一方阵、建设河南省新兴副中心城市的黄金机遇期。综合判断，“十三五”时期，开封发展大有作为、大有可为，但也面临着矛盾叠加、风险隐患增多的严峻挑战。我国经济进入新常态，长期向好的基本面没有改变，但结构性产能过剩严重，中央提出“三去一降一补”五大任务，大力推进供给侧结构性改革，将全面启动市场需求，激发经济发展新动力。新一轮科技革命和产业变革蓬勃兴起，新技术新产品新成果转化带来新的增量，“双+”“双创”不断催生发展新活力。中央将出台中部崛起新十年计划，省委、省政府强力推进中原经济区和郑州航空港经济综合实验区建设，着力构建“一极三圈八轴带”发展格局，大力支持开封建设河南省新兴副中心城市，打造郑汴一体化升级版，必将释放新的发展潜力。经过多年的积累和探索，我市发展思路清晰坚定，综合实力不断增强，发展基础更加坚实，并形成了一套行之有效的体制机制，全市上下精诚团结、信心倍增，干事创业热情高涨，晋位升级、跨越发展的正能量正在汇聚。我们必须凝聚全市人民的智慧和力量，务实拼搏，砥砺前行，向全面建成小康社会目标奋勇前进。

　　今后五年，我市经济社会发展的指导思想是：高举中国特色社会主义伟大旗帜，全面贯彻党的十八大和十八届三中、四中、五中全会精神，以马克思列宁主义、毛泽东思想、邓小平理论、“三个代表”重要思想、科学发展观为指导，深入贯彻习近平总书记系列重要讲话精神，按照“五位一体”总体布局和“四个全面”战略布局，坚持发展是第一要务，牢固树立和贯彻落实创新、协调、绿色、开放、共享发展理念，围绕建设河南省新兴副中心城市总目标，强化新型工业化城市和国际文化旅游名城定位，抢抓“一带一路”战略、中原经济区与郑汴一体化发展、郑州航空港经济综合实验区建设重大机遇，着力优化经济结构，着力转换发展动力，着力厚植发展优势，着力保障和改善民生，建设实力开封、文化开封、美丽开封、幸福开封，力争经济实力进入全省第一方阵，确保如期全面建成小康社会。

　　今后五年，我市经济社会发展的主要目标是：建设实力开封。生产总值年均增长9%左右，一般公共预算收入年均增长10%左右，经济增长速度保持全省领先水平。城镇居民和农村居民人均可支配收入与经济增长同步，分别达到3.7万元和超过1.8万元。三次产业结构调整为12∶43∶45。城镇化率达到56%。建设文化开封。公共文化基础设施建设全面达到国家规定标准，覆盖城乡的公共文化服务体系基本健全。宋都古城文化产业园区特色彰显，国际文化旅游名城影响力不断增强。“文化+”行动取得显著成效，文化产业增加值占生产总值的比重达到7%，文化发展实力居全省前列。建设美丽开封。资源节约型和环境友好型城市建设取得新成效，主要污染物排放总量明显减少，大气和水质量达标率明显提高，生态安全屏障基本形成，林木覆盖率达到23%，城市建成区绿地率达到40%，自然生态人居环境明显改善。建设幸福开封。每年投入民生资金占公共预算支出比例不低于70%，社会事业全面进步，社会保障日趋完善，社会养老服务体系加快建立，居民生活质量、健康水平、居住条件明显提升，人民群众安全感、幸福感、获得感持续增强。

　　今后五年，我市经济社会发展的战略导向是：始终贯彻“五大理念”：把创新作为引领发展的第一动力，把协调作为持续健康发展的内在要求，把绿色作为永续发展的必要条件，把开放作为繁荣发展的必由之路，把共享作为加快发展的本质要求。实施重点战略举措：继续实施工业强市战略，推动工业经济提质增效；着力推进服务业发展，建设国际文化旅游名城；建设“四带三区”，打造郑汴一体化升级版；突出“两核六组团”，促进城乡协调发展；推进“双+”“双创”，培植发展新优势；加快对外开放步伐，构建改革开放新平台。

　　各位代表！

　　《开封市国民经济和社会发展第十三个五年规划纲要（草案）》及说明已印发各位代表，请与《政府工作报告》一并审议。

　　我们坚信，有省委、省政府和市委的坚强领导，有市人大、市政协的鼎力支持，有550万开封人民的奋勇拼搏，我市“十三五”发展蓝图一定能够如期实现！“四个开封”、河南省新兴副中心城市建设一定能够再奏凯歌、再谱新篇！

　　三、切实做好2016年工作，努力实现“十三五”良好开局

　　2016年是全面建成小康社会决胜阶段的开局之年，也是推进结构性改革的攻坚之年，做好今年各项工作，意义重大。

　　2016年我市经济社会发展主要预期目标是：生产总值增长9%左右，一般公共预算收入增长10%左右，规模以上工业增加值增长10%，全社会固定资产投资增长15%，社会消费品零售总额增长12%，进出口总值增长7%，实际到位省外资金增长10%，城镇新增就业5.5万人以上，常住人口城镇化率提高1.5个百分点左右，城乡居民收入增长与经济增长基本同步，居民消费价格涨幅3%左右，节能减排、环保等约束性指标完成省下任务，发展质量和效益明显提高，结构性改革取得实质性进展。

　　完成今年目标任务，必须做到“三个统一”，抓住“五个重点”，即：把认识和行动统一到中央对经济工作的准确判断上来，做到方向明；把责任和力量统一到省委、省政府对开封的定位和厚望上来，做到发力准；把目标和举措统一到我市“十三五”规划的总体部署上来，做到措施实。围绕“经济增长点”抓培育，围绕“精准发力点”扩增量，围绕“加快发展关键点”上项目，围绕“重要支撑点”强保障，围绕“共享发展落脚点”惠民生。重点抓好以下十个方面的工作：

　　（一）培育新的经济增长点。启动“开港经济带”建设，着力培育战略性新兴产业和现代物流业。紧密对接郑州航空港经济综合实验区，坚持高端谋划，高标准建设，确保成功申报国家产城融合示范区。加快开港大道建设，确保2017年7月竣工通车。有序展开产业布局，着力引进高附加值、高科技含量企业，重点发展高端制造业和现代物流业，加快已布局项目建设。以开港经济区为依托，积极申报保税物流中心。推进“示范区CBD”建设，着力培育现代服务业。完善基础设施和商业配套服务体系，确保5月底中意湖蓄水，年底前博物馆、规划馆、美术馆建成开放，在建的CBD商务楼宇群力争交付使用。依托已获批的国家级电子商务示范基地，新培育50家电子商务企业。加快绿地中部创客天地项目建设，搭建创客集聚平台、研发支撑平台，再建一批众创空间，打造“双创”活力地带。大力培育现代金融新业态，做大做强金融业。加快大河印象文化城等项目建设，积极发展文化创意产业。推进“宋都古城文化产业园区”建设，全面提升文化旅游业。加快宋都文化商务区、城摞城新郑门遗址、地下城墙展示馆、小宋城二期、艮岳宋代园林博物馆等项目建设。建成中原明珠文化旅游产业园、建业东京梦华半亩园、双龙巷历史街区改造等项目，启动清明上河园三期、建业泰和府、非遗创意园等项目建设，启动大相国寺周边综合改造，完成马道街街景整治。设立文化旅游产业发展基金，加强对文化旅游企业引导扶持。办好清明文化节、菊花文化节、收藏文化论坛和国际茶文化研讨会。以创建全国旅游标准化示范城市为契机，加快旅游集散中心建设，完善旅游基础设施，加大开封旅游整体宣传推介，促进文化旅游业升级发展。启动“东部新城”建设，着力培育新能源新材料产业。高标准编制总体规划，启动以祥符区城区为核心的东部新城建设，谋划好新城发展和产业布局，力争核心城区“一年一个样、三年大变样、五年成规模”。做好祥符区与老城区基础设施对接共享，贯通华夏大道至祥符区，逐步完善配套基础设施。做大做强主导产业，重点培育新能源新材料、高端化工、精细化工产业，夯实东部新城产业基础。打造“郑汴一体化升级版”亮点，着力培育产学研和大健康产业。启动郑汴产学研结合示范带、沿黄生态带规划编制和建设工作。完善职业教育园区基础设施，开工建设省外贸学校、军威科技职业学院、化工职业学院，力争再筹建一所工科类学校。加强职业院校与企业合作，推动各类科研成果和实用技术转化应用，布局延伸产学研产业。积极发展大健康产业，确保中心医院、体育中心等项目开工建设，做大做强生物医药和高端医疗器械制造等产业，积极培育健康养老、养生休闲、体育消费等新兴业态。以实施“双+”“双创”为契机，催生新业态新模式。落实好“双+”行动计划、“双创”工作意见，用好“文化+”发展资金，开展“文化+”十大专项行动。实施“513”工程，即实施文化旅游、商贸流通、健康养老、房地产、“文化＋”5大类超过100个服务业重点项目，力争全年投资达到300亿元。创新“互联网+”新模式，着手实施数据整合工程，提升城市智能化水平。继续实施科技企业孵化器发展三年行动计划，推广创客空间、创新工场等新型孵化模式，打造创新创业生态全链条。

　　（二）促进工业经济提质增效。积极推进供给侧改革。出台扶持工业经济发展的政策措施，稳定优质存量，持续扩大有效增量。深入开展服务企业活动，研究制定“降本增效”跟进措施，切实减轻企业负担。对长期亏损、资不抵债、停产半停产的“僵尸企业”实行兼并重组，妥善处置。支持研发新产品。用好5000万元工业转型升级专项基金，引导企业加快新技术转化和新产品研发。加快奇瑞汽车SUV新车型、通海流体核电级专用阀门等新产品研发进度。实施质量兴市战略，实现省长质量奖“零突破”。开展“文化+工业”专项行动，加快汴绣文化研发基地等项目建设，推动新型工业化与“文商旅”模式融合发展。加快转化新技术。支持“双50”企业建设高水平技术研发中心，确保国家空分设备质量监督检验中心建成投用。推进开封炭素、开封仪表等企业技术升级改造，加快新技术成果转化。大力实施新项目。严格按时间节点推进重点项目“321”工程，即实施300个以上重点项目，总投资超过2000亿元，全年完成投资超过1000亿元，着力抓好32个省级A类重点项目。实施“7131”工业项目工程，围绕7大类产业实施150个以上工业项目，力争完成工业项目投资300亿元，带动全市工业投资增长10%以上。重点推动易成新能负极新材料、优德医疗设备等一批重大工业项目建设。完善产业体系。调整产业发展重点，依托产业集聚区和服务业“两区”，发展壮大装备制造及汽车和零部件、农产品加工、化工、纺织服饰等4个传统产业集群，积极培育新能源新材料、电子信息、生物医药等3个新兴产业集群，着力推动文化旅游、商贸物流、健康养老3个服务业产业集群发展，构建“4+3+3”产业体系，加快培育百、千亿级优势产业集群。突出整产业链发展，加快产业集聚区建设，努力形成一批有特色、有规模、具有核心竞争力的特色专业园。

　　（三）推动服务业转型升级。壮大服务业企业规模。落实好服务业发展指导意见，发挥3000万元服务业引导基金作用，推动商贸流通、特色餐饮等传统服务业转型升级。大力发展现代金融、教育培训、科技服务等高成长性服务业。实施消费产品升级工程，加快培育百家名店、百家老字号。鼓励服务业企业抱团发展、集聚发展，力争入规入库服务业企业300家。加快中心商务区、特色商业区建设。把发展重心转到促集聚、育特色、提服务、强融合上，打造10个以上主营业务收入超亿元的服务业“两区”和特色市场。加快开封国际农业会展中心等42个投资亿元以上“两区”重点项目建设，力争全市服务业“两区”固定资产投资完成100亿元，主营业务收入增长15%以上。培育新的消费增长点。启动实施新兴热点消费促进工程，研究刺激消费政策措施，搞活以互联网为载体、线上线下互动的新兴消费，促进养老家政健康消费，扩大教育文化体育消费，持续提升农村消费能力。大力发展电子商务，全市新增电商企业、电商商户1000家以上。促进房地产业健康发展。落实好促进房地产业健康发展各项政策措施，完善进城农民购房激励政策，引导房地产企业顺应市场规律，合理确定商品房价格。统筹保障性住房和商品房建设，探索施行收购存量商品住房用于保障房政策，打通商品房、安置房、保障房转换渠道，加快消化房地产库存。

　　（四）统筹推进农业农村工作。打好扶贫攻坚战。按照扶持对象精准、项目安排精准、资金使用精准、措施到户精准、因村派人精准、脱贫成效精准的要求，“转、扶、搬、保、救”五措并举，实施扶贫攻坚“五个一批”工程，完成扶贫开发整村推进村50个，再实现3.5万农村贫困人口稳定脱贫。稳定提高粮食产量。实施藏粮于地、藏粮于技战略，推进高标准粮田建设，确保粮食总产实现“十三连增”。加快农业科技创新及成果转化，提高农业综合机械化率，持续推进气象防灾减灾和为农服务体系现代化建设。持续抓好灌区续建配套与节水改造、中小河流治理、病险水闸加固等水利工程建设，力争每县启动建设一处引黄调蓄工程，新增改善有效灌溉面积26万亩。打通农产品全产业链。树立大农业、大食物观念，推进农村一二三产业融合发展。围绕生猪、肉鸡、肉牛、大蒜等优质农产品，重点打通9条农产品全产业链，支持牧原、天丰、大用、包公食品等农业产业化龙头企业发展壮大，新培育现代农业产业化集群3个，力争全市农产品加工业增加值超过400亿元。探索多种交叉融合方式，培育农村电商、农产品定制等“互联网+”新业态。实施都市生态农业发展工程，选择6个有条件的乡镇，积极发展休闲观光农业，拓宽农民就业增收空间。持续深化农村改革。基本完成农村土地承包经营权确权登记颁证工作，全面启动农村集体建设用地使用权确权登记发证工作，出台引导农村土地经营权有序流转实施方案。落实农业“三项补贴”改革，发展种粮大户、家庭农场等新型农业经营主体，健全农业公共服务和社会化服务体系，发挥农业适度规模经营在结构性改革中的引领作用。

　　（五）促进城乡协调发展。协调“两核六组团”发展。“两核”定位：老城区以城墙内13平方公里为核心，以宋都古城文化产业园区为载体，以宋文化为主题、文商旅融合为重点，努力建设成为全城一景、宋韵彰显的国际文化旅游名城目的地。示范区以CBD为核心，以开港经济带、郑汴产学研结合示范带、沿黄生态带为载体，以发展现代服务业、装备制造及汽车和零部件产业为重点，实施二次创业，开展第二个五年攻坚，全力打造郑汴一体化升级版，努力建设成为河南省新兴副中心城市的核心增长极。“六组团”定位：东部新城组团以祥符区城区为核心，祥符区、顺河回族区、禹王台区多组团协调发展，以黄龙、汴东、精细化工产业集聚区为载体，以发展新能源新材料、精细化工、高端化工为重点，努力建设成为区域协调发展的东部新城。朱仙镇组团以朱仙镇古镇为核心，依托开港经济带重要节点优势，以朱仙镇文化旅游发展试验区为载体，以文化体验、休闲度假、健康养老、科技教育为产业重点，努力建设成为享誉全国的历史文化古镇。尉氏县组团以建设全省经济综合实力“20强县”为总目标，强化郑汴一体化南部经济重心、中原经济区产城融合样板、郑州航空港经济综合实验区东部创新高地“三个定位”，以纺织服饰、健康医疗、现代家居为产业重点，努力建设成为全市县域经济发展的“排头兵”。杞县组团以打通南部经济带，开通郑民高速二期为契机，主动对接市区、联接港区，在持续壮大新能源新材料产业的同时，打造“粮经饲”统筹、“种养加”一体全产业链条，努力建设成为一二三产融合的示范区。通许县组团以打通南部经济带为契机，依托交通区位优势，主动对接市区、联接港区，深挖产业潜力，瞄准高端、高质、高新，以中低速电动汽车、电动特种车转型升级为重点，努力建设成为制造业转型升级的先行区。兰考县组团充分发挥省直管优势，建设地区副中心城市。加快中心城市建设。尊重城市发展规律，坚持人民城市为人民，走出一条具有开封特色的城市发展道路。高度重视古城保护和历史文脉延续，完成城市风格规划设计，加强对建筑设计、城市风貌的约束和指导。继续做好“两改一建”工作，实施24个棚户区、城中村改造项目，征收房屋7798户、183.6万平方米，解决33个棚户区、城中村改造项目及73个保障性住房项目遗留问题。开展精准化房屋征收，该征收的坚决征收到位，不留后遗症，能保护的坚决保护，避免大拆大建。巩固国家卫生城市、园林城市创建成果，以创建全国文明城市为载体，提高城市精细化管理水平，加强市民文明教育，市民素质高一分，城市形象美十分，让每一位开封市民都成为城市文明形象代言人。进一步改善城乡面貌。启动提升县级城市管理水平三年行动计划。继续开展“清洁家园·美丽乡村”和小城镇综合整治提升工程，推进原“133工程”连点、串线、扩面，实施新“133工程”，再建设100个美丽乡村、30个示范乡镇和30个活力社区，着力改善农村人居环境。加强指导，分类推进，妥善解决87个新型农村社区遗留问题。加快农业转移人口市民化。按照“一基本两牵动三保障”总体要求，推进以满足新市民为出发点的住房制度改革，推动户籍制度改革和居住证制度“双落地”。加强城乡社会保障制度衔接，维护好进城农民土地承包权、宅基地使用权、集体收益分配权。

　　（六）完善以“八网”为重点的基础设施建设。完善路网。重点完善城铁、高铁、高速路、快速路、干支线路、城市道路和公交线路，提高道路通行能力。启动郑开城际铁路延长线项目建设；建成郑开城际铁路宋城站、郑徐高铁开封北站广场及配套工程；确保郑民高速二期建成通车，继续推进机西高速二期（开封段）建设，完成郑民高速南苑站扩建工程，开工建设连霍高速开港大道出入口，做好连霍高速开柳路出入口前期工作；启动开尉路、开杞路改建和祥港大道建设工程；完成“农村公路三年行动计划”。全面建成2015年已开工道路。以打通断头路为重点，新开工建设孝严寺街（内顺城路-孝严寺桥）、夷山大街土城段（万胜路-电视塔南路）、内顺城路（龙亭北路西口-万岁山南口）、龙亭西路北延（万岁山南口-西关北街-东京大道）、东外环路北延（东京大道-复兴大道）、魏都路东延（五一路-中山路）等6条道路。继续大力发展公共交通，调整优化15条公交线路，加快宋城客运枢纽站、小李庄公交首末站等公交场站建设。完善水网。重点完善水系、引黄水利工程、雨污水管网、供水管网。开工建设涧水河工程，改扩建水系一期，启动建设开封西湖二期、碧水河、秀溪河、王庵水库、运粮河调蓄工程、引黄入杞工程。做好龙亭湖、包公湖、黑池清淤，治理黄汴河（东京大道孙李唐-滨河路皮革厂桥）、东护城河（东京大道仁和屯-滨河路皮革厂桥）、东干渠（建设路-化肥河）、化肥河、惠济河（侯庄桥-文庄桥）、北郊沟（发源地-东京大道仁和屯）6条黑臭水体。启动老城区第三污水处理厂、示范区第二污水处理厂建设，新建东京大道西外环路雨水泵站及东外环路、夷山大街北延污水管网。积极争取南水北调工程入汴，增加新的城市水源。完善热网。重点加快热源和配套管网建设，积极推进东区供热，市区新增供热面积350万平方米，新增供热家庭1.8万户，比2015年翻一番。完善气网。重点加强天然气配套管网建设，提高燃气应急保障能力。新建燃气管线77公里，市区燃气气化率达到90%以上。完善电网。加快22个110千伏及以上输变电工程建设，新建35千伏及以下供电线路600千米，提升全市总供电能力到354万千瓦。完善光网。全面推进宽带光纤化，城乡无线宽带速率提高到150M。新建50万光纤宽带端口，家庭宽带用户宽带接入能力达到20M以上。完善绿网。重点加强生态廊道、湿地、公园绿地及道路河渠两侧绿化，提升生态廊道绿化水平。实施惠济河湿地、马家河湿地综合保护工程，涵养“城市绿肺”。提升植物园绿化水平，开工建设环城墙公园六期（明伦街-汴京公园北围墙）、七期（地震台-万岁山），建设城市绿带。完善云网。积极整合政务云、企业云、旅游云、文化云等云端资源，加快智慧城市建设，建成大数据中心和智慧开封展示体验中心，启动应急指挥中心建设，力争再开工一批智慧项目。

　　（七）加强支撑保障体系建设。继续推进改革。深化行政体制改革，持续推进投资审批、收费清理、商事制度等改革。加快行政审批四级便民服务体系和标准化建设，建成30个“市民之窗”便民自助服务平台。深化国有企业改革，完善国有企业外派监事会制度，合理界定国有企业功能类别，实施分类改革、发展和监管。全面清理限制非公有制经济发展的不合理规定，促进非公有制经济持续快速健康发展。深化财税金融和投融资体制改革，推进消费税改革和资源税费改革，做好国税、地税征管体制综合改革。加快县域农信社组建为农商行，积极引进港中旅银行、郑州银行、中信银行等股份制银行，争取浦发银行档案中心、邮储银行办公中心、中原银行培训中心等后台服务中心落户开封。创新PPP项目收益和分配机制，完善公共产品价格形成机制。统筹抓好其它领域改革。实施创新驱动。激发企业、科研机构和高校、市场转化科技成果潜力。发挥“双+”、“双创”乘数效应，新培育高新技术企业5家、工程技术研究中心10家、市级重点实验室2~3家。加强新型研发机构、科技研发基地合作平台建设，新建众创空间、孵化器10家，培育科技型中小微企业100家。保护生态环境。深入实施蓝天、碧水、乡村清洁等工程，留住“碧水蓝天”。继续推进燃煤锅炉清洁能源改造或拆除，加大黄标车淘汰力度，加强城区运输车辆及施工工地扬尘污染治理。加大秸秆禁烧力度，做好重污染天气应急应对，遏制雾霾天气高发。继续实施水源地环境综合整治六大工程，强化地表和地下水污染防治，保障饮用水安全。继续实施城市清洁河流行动计划，强力推进城市排污治理。持续推进农业面源治理、土壤污染治理和林业生态市建设，力争创建4个省级生态乡镇和18个省级生态村。强化要素保障。坚持最严格的耕地保护制度，坚守耕地红线。更加注重土地挖潜，促进土地集约、节约利用。对批而未征、征而未供、供而未用的项目用地依法分类处理，达到收回条件的坚决予以收回，对因政府原因造成征收慢、供地慢的行为坚决查处。创新金融产品，扩大信贷投放，引导外资和民间资本采用PPP模式参与城市建设，用好已签约的300亿元城市发展基金。积极推进企业上市，实现7家企业上市融资。实施最严格的环境保护制度，建立健全覆盖所有固定污染源的企业排放许可、评价考核与责任追究制度，严厉打击环境违法行为，为发展留足环境容量。继续实施全民技能振兴工程，完成各类职业技能培训13.8万人次。加强市校合作，推进招才引智“2231”人才工程，提升人力资源保障能力。

　　（八）提升开放带动水平。深度融入“一带一路”。发挥“一带一路”城市旅游联盟作用，加强与国内外城市合作交流，携手推动更大范围、更高水平、更深层次的开放。鼓励企业走出去，支持空分、化工、农产品加工、空间结构等企业开拓海外市场。积极发展跨境电子商务。突出抓好开放招商。以十大产业集群和新技术、新业态、新模式招商为重点，以延链补链强链为方向，着力抓好具有引领性、突破性、方向性的重大项目引进。用好招商引资“六项机制”，强化三个招商链条，紧盯重点区域精准招商、联合招商、整体招商，鼓励现有企业增资扩股实现零地招商，力争全年引进亿元以上项目130个以上，实际到位省外资金增长10%以上，实际利用外资增长9%以上，新批千万美元以上外资项目20个。强化跟踪服务，提高招商实效，2014年和2015年签约的318个项目开工率达到50%以上。建好用好招商平台。充分发挥四个招商基地作用。全力做好河南自贸区（开封片区）申报工作，争取在开封设立海关、商检、跨境电子商务基地等机构，推动上海自贸区“28+6”经验复制落地。

　　（九）持续保障和改善民生。继续实施十项民生工程，办好为民实事。推动更高质量就业。实施更加积极的创业就业政策，进一步健全公共就业服务体系，扎实开展公共就业服务活动，打造全方位、立体化的就业服务网络，确保城镇新增就业5.5万人以上，农村劳动力转移就业3万人。提升社会保障水平。全面实施全民参保登记计划，推动社会保障由制度全覆盖转向法定人员全覆盖，确保城镇职工、城乡居民养老金按时足额发放。统筹推进城乡社会救助体系建设，完善最低生活保障、特困人员供养、残疾人帮扶等兜底性制度。健全关爱“三留守”人员工作机制。积极发展残疾人事业和慈善事业。努力办好人民满意的教育。科学规划，合理布局，完成第二期学前教育三年行动计划，新建、改扩建幼儿园44所，扶持普惠性民办幼儿园40所。深入推进全面改善义务教育薄弱学校基本办学条件和扩充城镇义务教育资源五年规划，新建中小学校6所、改扩建12所，促进义务教育均衡发展。推动普通高中内涵特色多样化发展。加快各类职业教育发展水平。支持河南大学、黄河水利职业技术学院等高校发展，办好开封大学、开封文化艺术职业学院。完善助学资助体系，让更多贫困家庭孩子有机会接受更好教育。提高人民健康水平。强化基层卫生服务能力建设，推进公共卫生服务均等化。做好重大传染病防控和公共卫生事件应急处置。支持中医药事业发展。实施全面“两孩”政策，改革完善计划生育服务管理。广泛开展全民健身运动，建成城墙体育公园，新建240个农民体育工程、46个健身路径工程。提高体育竞技水平，办好2016中国郑开国际马拉松赛和中国（开封）客家国际龙舟邀请赛。扩大基本公共文化服务。继续实施文化惠民全覆盖工程，组织开展“舞台艺术送农民”、政府购买公益性演出、电影公益放映活动。加快推进公共文化基础设施建设，高标准建成市美术馆，启动市图书馆、群艺馆新馆前期工作。拓展公共文化服务场所免费开放功能，大力倡导全民阅读，丰富群众精神生活。加强非物质文化遗产的传承、保护和利用，积极推进开封城墙申报世界文化遗产。防范和化解各类风险。严格落实安全生产责任制，坚决防范和遏制重特大安全事故发生。完善食品药品安全监管体制机制，防止食品药品安全事件发生，积极创建全国食品安全示范城市。落实属地管理责任和监管部门责任，集中解决非法集资问题，最大限度维护群众利益、维护经济发展、维护社会稳定。完善社会治理。健全社会治安立体防控体系，完善反恐和打击犯罪机制，强化流动人口和特殊人群服务管理，落实社区矫正工作。做好信访维稳工作，更加注重依法化解社会矛盾。加强社区建设，推行政府购买社区社会服务试点。加强互联网舆论引导与管理，努力打造清朗网络空间。

　　继续加强国防动员、国防教育和国防后备力量建设，支持开封驻军、武警部队、预备役部队、民兵和人民防空建设，深入开展双拥共建，巩固军政军民团结，推动军民深度融合发展。支持工会、共青团、妇联等人民团体更好发挥桥梁纽带作用，注重发挥工商联等社会团体作用。依法管理民族宗教事务，大力发展民族经济，促进民族团结进步、宗教关系和谐。加强统计、督查工作。继续做好外事、侨务、港澳、对台、史志、地震、档案、社科研究等工作。

　　（十）打造人民满意的政府。打铁还需自身硬。今年我们将以更大的决心、更实的措施，持续推进法治政府、服务政府、廉洁政府、责任政府建设。坚持依法行政。牢固树立法治理念，运用法治思维和法治方式解决问题，按照法定权限和程序行使权力、履行职责。坚持依法科学民主立法，推进市容环境卫生、饮用水水源保护等立法。健全依法决策机制，完善公众参与、专家论证、风险评估、合法性审查等重大行政决策法定程序。强化依法治理，启动“七五”普法。推进服务型行政执法，促进严格规范公正文明执法。转变工作作风。持续践行“三严三实”，驰而不息纠正“四风”，健全岗位履职责任和激励机制，健全全面覆盖的督查机制，完善效能问责办法，解决“中梗阻”，让那些身在岗位不作为、拿着俸禄不干事、占着位置不担当、庸政懒政怠政的政府公务人员绝无立身之地。强化责任担当。保持昂扬向上、积极进取精神状态，面对矛盾困难敢于迎难而上，面对深层次难题敢于知难而进，做到守土有责、奋发有为，政府提出的规划目标、发展任务、民生实事、作出的承诺，做到事事有时间表，人人有任务书，步步有路线图，说了算，定了干，干到成。依法接受监督。强化行政权力制约和监督，加强和改善事前事中事后监管，全面推进政务公开。依法接受人大的法律监督和工作监督，认真落实市人大及其常委会的各项决议决定，坚持政府重大决策向人大报告制度。主动加强同人民政协的联系，积极支持市政协履行政治协商、民主监督和参政议政职能，认真办理人大代表建议和政协提案。保持清正廉洁。始终把纪律挺在前面，认真履行主体责任和“一岗双责”，严格落实党风廉政建设责任制，严格执行廉政准则和各项制度规定，强化行政监察和审计监督，坚决查处权力寻租、权钱交易和损害群众利益的行为，以零容忍态度惩治腐败，以反腐倡廉的实际成效保障发展、取信于民！

　　各位代表！

　　人民群众的期待就是我们努力的方向！人民群众的所需就是我们奋斗的目标！当前，开封的发展已经站在新的历史起点上，前景令人鼓舞，梦想催人奋进。让我们紧密团结在以习近平同志为总书记的党中央周围，高举中国特色社会主义伟大旗帜，在省委、省政府和市委的坚强领导下，紧紧依靠和团结带领全市人民，继续务实苦干，奋勇拼搏，努力把“十三五”规划的宏伟蓝图变为现实，让无限的希望和梦想变为现实，为加快建设实力开封、文化开封、美丽开封、幸福开封，加快建设河南省新兴副中心城市，全面建成小康社会而努力奋斗！
